# Gut microbiota response to antibiotics is personalized and depends on baseline microbiota

**DOI:** 10.1186/s40168-021-01170-2

**Published:** 2021-10-27

**Authors:** Armin Rashidi, Maryam Ebadi, Tauseef Ur Rehman, Heba Elhusseini, Harika Nalluri, Thomas Kaiser, Shernan G. Holtan, Alexander Khoruts, Daniel J. Weisdorf, Christopher Staley

**Affiliations:** 1 Division of Hematology, Oncology, and Transplantation, Department of Medicine, University of Minnesota, 14-100 PWB, MMC 480, 420 Delaware St. SE, Minneapolis, MN 55455 USA; 2 Division of Gastroenterology, Hepatology, and Nutrition, Department of Medicine, University of Minnesota, Minneapolis, MN USA; 3 Department of Surgery, University of Minnesota, Minneapolis, MN USA

**Keywords:** Antibiotics, Leukemia, Microbiota

## Abstract

**Background:**

The magnitude of microbiota perturbations after exposure to antibiotics varies among individuals. It has been suggested that the composition of pre-treatment microbiota underpins personalized responses to antibiotics. However, this hypothesis has not been directly tested in humans. In this high-throughput amplicon study, we analyzed 16S ribosomal RNA gene sequences of 260 stool samples collected twice weekly from 39 patients with acute leukemia during their ~ 4 weeks of hospitalization for chemotherapy while they received multiple antibiotics.

**Results:**

Despite heavy and sustained antibiotic pressure, microbial communities in samples from the same patient remained more similar to one another than to those from other patients. Principal component mixed effect regression using microbiota and granular antibiotic exposure data showed that microbiota departures from baseline depend on the composition of the pre-treatment microbiota. Penalized generalized estimating equations identified 6 taxa within pre-treatment microbiota that predicted the extent of antibiotic-induced perturbations.

**Conclusions:**

Our results indicate that specific species in pre-treatment microbiota determine personalized microbiota responses to antibiotics in humans. Thus, precision interventions targeting pre-treatment microbiota may prevent antibiotic-induced dysbiosis and its adverse clinical consequences.

Video abstract

**Supplementary Information:**

The online version contains supplementary material available at 10.1186/s40168-021-01170-2.

## Background

**Fig. 1:**
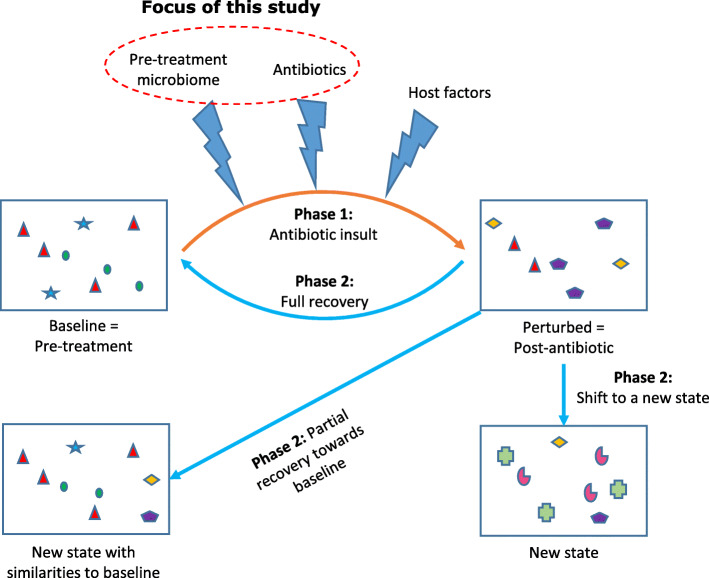
Phases of microbiota response to antibiotics. After antibiotic exposure, baseline microbiota undergoes an initial perturbation (phase 1), during which some taxa may completely disappear, while some new taxa may arise. Potential determinants of phase 1 include baseline microbiota, specifics of antibiotic exposure, and host factors (e.g., genetics and immunity). After completion of antibiotic treatment, the microbiota undergoes reorganization (phase 2), which could lead to full recovery to baseline, partial recovery towards baseline, or shift to a completely new state. The focus of this study is the role of baseline microbiota in regulating phase 1 after controlling for antibiotic exposures

Antibiotics represent a major cause of perturbation experienced by microbial communities in the body. The typical progression of the compositional states of microbiota after antibiotic-induced perturbations includes departure from baseline (phase 1) followed by post-antibiotic reorganization (phase 2) (Fig. 1). Reorganization of microbiota composition after cessation of antibiotics may lead to full recovery to the original pre-treatment state. Alternatively, recovery may be partial, with the new community having some degree of similarity to pre-treatment microbiota. Yet in other cases, reorganizing microbiota can shift to completely new states with little resemblance to the baseline community [1–3], commonly referred to as a “regime shift” [4, 5]. A resistant community can be defined as one that resists perturbation, while a resilient community is one that recovers after perturbation [6]. A resilient community will likely stabilize into a fully functional state after transitioning through phases of perturbation and recovery [7, 8]. Unresolved disruptions in microbiota lead to loss of colonization resistance [9], deleterious health consequences such as *Clostridioides difficile* infection [10], and altered metabolic functions important for host food processing [11], drug metabolism [12], and endocrine homeostasis (e.g., appetite regulation) [13].

Microbiota response to antibiotics varies among individuals [2] and the mechanism of response is poorly understood [1, 14]. Intuitively, characteristics of the antibiotic insult (e.g., antibiotic type and duration), composition and wiring of pre-treatment microbiota, and host factors (e.g., genetics and immunity [15]) are possible determinants of individualized microbiota responses. Here, we hypothesize that pre-treatment microbiota in humans is a determinant of community resistance as measured inversely by the magnitude of microbiota deviation from baseline during phase 1. Pre-clinical data support this hypothesis. Germ-free mice humanized with fecal microbiota from different donors show different microbiota responses to the same antibiotic [16], suggesting that pre-treatment microbiota may underpin individualized responses. In addition, the bloom of specific pathogens after antibiotic treatment has been associated with the initial state of the microbiota [17]. However, it is unknown whether and to what extent specific species in baseline microbiota modulate community resistance against antibiotic perturbation.

Antibiotic exposure may be light and brief (e.g., one dose of one antibiotic), light and sustained (e.g., several-day course of one antibiotic), heavy and brief (e.g., brief course of multiple antibiotics), or heavy and sustained (e.g., prolonged course of multiple antibiotics). We studied patients with acute myeloid leukemia (AML) receiving inpatient chemotherapy, a prototype setting for heavy sustained antibiotic exposure. These patients receive multiple antibiotics over several weeks of hospitalization. Initially, patients receive antibiotics to prevent infections. However, most patients develop a fever despite prophylaxis [18]. At the time of fever, prophylactic antibiotics are escalated to broader-spectrum antibiotics, which are later adjusted according to the results of microbiological work-up. These antibiotics are de-escalated to prophylactic antibiotics or discontinued as clinical and hematopoietic recovery ensue. This cascade of events can be summarized as overlapping waves of antibiotic exposure, sometimes including multiple antibiotics for extended periods.

High-throughput amplicon sequencing of longitudinal stool samples collected from hospitalized patients with AML coupled with granular antibiotic exposure data uniquely positioned us to measure the dependency of microbiota perturbations on the microbiota’s baseline state. We find that the composition of pre-treatment microbiota is a major determinant of the magnitude of microbiota departure from baseline. We identify pre-treatment taxa that stabilize or destabilize the microbiota. These findings offer a mechanistic explanation for individualized responses to antibiotics and introduce novel targets for precision interventions to prevent antibiotic-induced dysbiosis and its adverse clinical consequences.

## Methods

### Patients and samples

We enrolled hospitalized adult patients with newly diagnosed or relapsed/refractory AML to a prospective, biorepository protocol (registration number in ClinicalTrials.gov: NCT03316456) approved by the University of Minnesota Institutional Review Board. An expected ~ 4 weeks of hospitalization was required. No other inclusion or exclusion criteria were used. Clinical and supportive care followed our standard institutional algorithm. Deviations per the treating physicians’ discretion were permitted. Our antibiotic stewardship recommends acyclovir for viral, an azole for fungal, and levofloxacin for bacterial prophylaxis for the duration of neutropenia. Bacterial prophylaxis is continued until development of neutropenic fever or first neutrophil rise above 1 × 10^9^/L, whichever occurs first. Cefepime is our recommended empiric frontline antibiotic for neutropenic fever. When oral intake decreases to < 60% of the lower limit of estimated energy and protein needs for 7 days, we generally initiate parenteral nutrition.

Stool samples were collected twice weekly (Mondays and Thursdays, ± 1 day window) between hospital admission and day 28 of chemotherapy or discharge (whichever occurred first). Collections were independent of clinical factors. Stool samples were collected in 95% ethanol-filled sterile tubes and stored at – 80 °C. Antibiotic exposure data were collected from electronic medical records for the following classes of antibacterial antibiotics: fluoroquinolones, 3rd or higher generation cephalosporins, piperacillin-tazobactam, carbapenems, metronidazole, oral vancomycin, and intravenous vancomycin.

### 16S ribosomal RNA (rRNA) gene sequencing

DNA was extracted using the DNeasy PowerSoil DNA isolation kit (QIAGEN, Hilden, Germany). The V4 hypervariable region of the 16S rRNA gene was amplified on an Illumina MiSeq platform (2 × 300 paired-end mode) by the University of Minnesota Genomics Center [19]. Sequences were processed in QIIME 2 [20]. Quality filtering, adaptor trimming, and stitching of raw sequences were done using the quality control pipeline SHI7 (trim threshold 32, threshold of Q37) [21]. Paired ends were merged using FLASH [22]. Operational taxonomic unit (OTU) picking was done using NINJA-OPS (default parameters and 97% similarity threshold) and the Greengenes database; Bowtie2 was used for alignment [23–25]. Species-level taxonomy was not considered given our short amplicon methodology. OTUs with a frequency of < 0.01% of reads and samples with fewer than 500 reads were removed. Sequence data were deposited in the Sequence Read Archive of the National Center for Biotechnology’s Information (NCBI) under BioProject ID SRP141394. The BIOM table was exported from QIIME into R 3.4 (R Foundation for Statistical Computing, Vienna, Austria).

### Microfluidic quantitative PCR for antibiotic resistance genes

Remaining DNA from a subset of samples was used for microfluidic qPCR (MFQPCR) to quantify various antibiotic-resistance genes (ARGs) including those for beta-lactams, quinolones, and vancomycin resistance as previously described [26]. BioMark HD System (Fluidigm) and 96.96 Dynamic Array IFCs (Fluidigm) were used for analysis. Serial dilutions (2 × 10^0^ to 2 × 10^6^ copies/μL) of the mixture of synthetic DNA fragments containing each target gene sequence were also included in the analysis to generate standard curves. Specific target amplification (STA) was done with 14 PCR cycles to increase the target DNA molecule prior to MFQPCR as previously described [27]. Sample DNA, gBlock standard mixtures, and no template controls were subjected to STA. Quantitative results obtained by MFQPCR were analyzed using Real-Time PCR Analysis software version 4.1.2 (Fluidigm) as described previously [27]. All samples were run in duplicate and their average gene content for each ARG (gene copies/microL DNA) was calculated.

### Statistical analysis

All analyses were performed using custom scripts and packages *phyloseq*, *vegan*, *lme4*, and *PGEE* in R. Linear discriminant analysis (LDA) coupled with effect size measurements (LEfSe) using patient number as “subject” was used to find taxa that differed significantly (LDA score > 3.0, *p* < 0.05) between the two groups [28]. OTU abundances were centered log-ratio (clr) transformed to account for the compositionality of microbiota. Dissimilarity between samples was quantified by Aitchison distance [29]. A pseudocount of min/2 was added to exact zeros prior to transformation. Alpha diversity was estimated by Shannon’s index [30]. We used Δm to denote Aitchison distance between non-baseline and baseline (pre-treatment) microbiota for the same patient, providing a single numerical value representing the magnitude of microbiota perturbation from baseline. Ordination was visualized by principal component analysis (PCA) and the proportion of variance explained by the individual was determined by permutational analysis of variance (PERMANOVA) with an adonis test (999 permutations) [31]. Other variables included in adonis were the first PCA axis of antibiotic history (see below), use of parenteral nutrition, and interval from baseline. Samples were the units of analysis and Δm was the outcome variable in all models unless specified otherwise. The performance of all models was estimated by Pearson’s correlation coefficient comparing observed vs. predicted values of Δm.

#### Quantification of antibiotic history

We quantified the “antibiotic history” of each sample using the time series of exposures to 7 major classes of antibacterial antibiotics between hospital admission and the day the sample was collected. The antibiotic classes considered were fluoroquinolones, third or higher generation cephalosporins, metronidazole, piperacillin-tazobactam, intravenous vancomycin (also daptomycin or linezolid), oral vancomycin, and carbapenems. For a given day, if a given antibiotic was used, it was coded 1 and otherwise, zero. Day 0 was defined as the first day of chemotherapy. Next, we applied a decaying average function to the time series of 0s and 1s for each antibiotic class to achieve a single numerical value summarizing exposure history for the given sample and antibiotic. As an example, if levofloxacin was used on days 1–3 for a patient admitted on day − 1 (1 day before starting chemotherapy), the time series for levofloxacin for a sample collected on day 5 of chemotherapy from this patient would be (0,0,1,1,1,0), indicating that the antibiotic was not used on days − 1, 0 (first day of chemotherapy), and 4, but was used on days 1, 2, and 3. With a decay factor of 2, the levofloxacin history for this sample would be quantified and summarized as 0 × 2^0^ + 1 × 2^−1^ + 1 × 2^−2^ + 1 × 2^−3^ + 0 × 2^−4^ + 0 × 2^−5^ + 0 × 2^−6^ = 0.875. With this decay factor, exposure on a given day receives twice as much weight than exposure on the previous day. A smaller decay factor would make the differences between the weights smaller. We ran Procrustes analysis [32] (999 permutations) to evaluate the correlation between microbiota composition (Aitchison distance) and antibiotic history (Euclidean distance); the sensitivity of this correlation to the decay factor was assessed.

#### Principal component linear mixed effect regression (package lme4, function lmer)

The goal of this model was to evaluate whether baseline microbiota predicts Δm after controlling for antibiotics and other covariates. Independent fixed effect variables included the following: the top principal components (PCs) of antibiotic history for each non-baseline sample, the top microbiota PCs for baseline microbiota, use of parenteral nutrition, interval from baseline, baseline Shannon index, and sample read depth. Patient ID was a random effect. *p* values were estimated from 200 bootstraps.

#### Penalized generalized estimating equations (PGEE) [33, 34]

While principal component regression allows us to understand the association between baseline microbiota and Δm after adjusting for covariates, it does not identify specific pre-treatment taxa that made the largest contribution to Δm. We implemented penalized GEE to analyze longitudinal data with many covariates [35]. The main idea of PGEE is that in models with many potential microbiota predictors, typically most taxa do not contribute to the outcome variable, and thus, regression coefficients for those variables should be zero. Using a penalty function, PGEE shrinks the estimates of small regression coefficients toward zero. A cutoff of 10^−3^ for shrunken coefficients is conventionally used to exclude variables from the model. Variables that remain in the final model are important predictors of the outcome variable, regardless of the specific value of their regression coefficients. The original model included 133 features: clr abundances of the OTUs collapsed at genus level (121 features) at baseline, decay averaged antibiotic histories (7 features representing 7 antibiotic classes), age, sex, body mass index, use of parenteral nutrition (categorical), baseline Shannon index, sample depth, and interval from baseline (days). Patient ID was a random effect. Fourfold cross validation (function *CVfit*) was used to estimate the optimal value of the tuning parameter, which was then used to build the final model (function *PGEE*). A first-order autoregressive correlation structure (i.e., observations closer to each other being more strongly correlated than those farther apart) was assumed. PGEE produces a consistent and asymptotically normal estimator even if the assumed correlation structure is misspecified. *p* < 0.05 was used to define statistical significance.

## Results

**Fig. 2:**
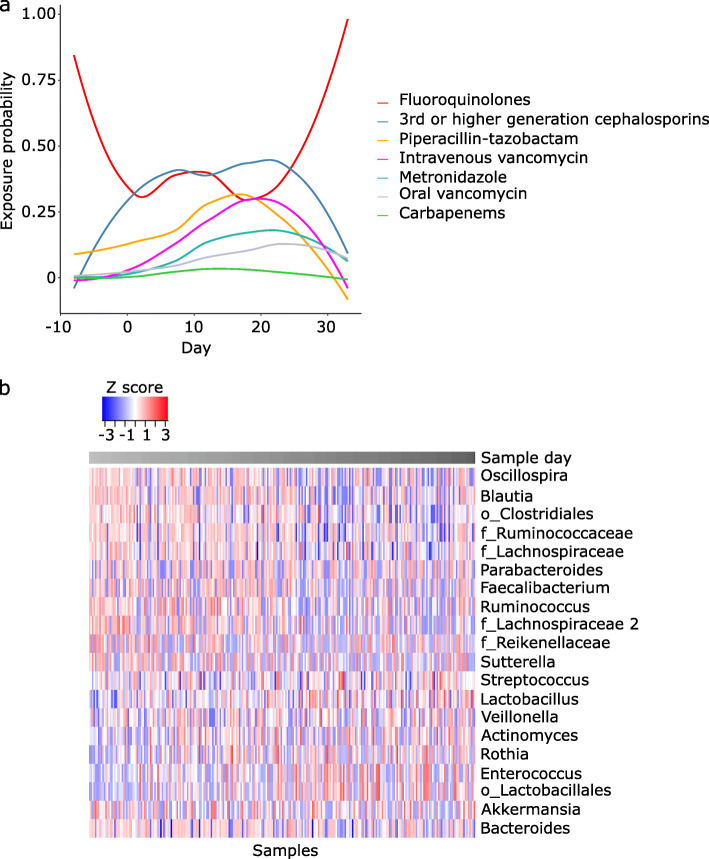
Antibacterial antibiotic exposures and microbiota heatmap. **a** Probability of exposure to each class of antibacterial antibiotics over time, with time measured in days and relative to the first day of chemotherapy. Linezolid and daptomycin were grouped together with intravenous vancomycin. Exposure for a typical patient starts with fluoroquinolone prophylaxis, followed by neutropenic fever, when fluoroquinolone is changed to broader-spectrum antibiotics (especially cefepime, piperacillin-tazobactam, and vancomycin); hence, there is an initial decline in the fluoroquinolone curve, but rises in other antibiotic curves. Antibiotic pressure peaks in weeks 2 and 3. Concurrent with clinical and hematopoietic recovery in week 4, broad-spectrum antibiotics are discontinued or de-escalated to prophylactic fluoroquinolones, the latter explaining the late rise in the fluoroquinolone curve. **b** Heatmap of the 20 most abundant genera. Each column indicates a sample, and each row represents genus-level mapping of an OTU. f (family) and o (order) indicate OTUs that were unclassifiable to deeper levels. OTU relative abundances were Z-transformed (indicated by the color gradient). Rows were ordered by their mean values and columns by sample collection day (color bar at top) relative to the first day of chemotherapy, with lighter colors indicating earlier days

Thirty-nine patients with AML provided twice-weekly stool samples during their ~ 4 weeks of hospitalization for chemotherapy. Fifteen patients required parenteral nutrition, and all but one patient developed a fever. As expected, antibiotic exposure was heavy and sustained (Fig. 2a). After filtering, we analyzed 260 samples (median of 7 per patient) containing an average of 19,604 high-quality reads per sample. A heatmap of the 20 most abundant genera is shown in Fig. 2b. Additional file 1: Figure S1a shows the aggregate relative abundance of the most abundant taxa in baseline and non-baseline taxa. Additional file 1: Figure S1b shows differentially abundant taxa in each group, adjusting for patient ID. The genus most prominently expanding from baseline to subsequent samples was *Enterococcus* (Additional file 1: Figure S1), consistent with our previous reports [36, 37].

**Fig. 3:**
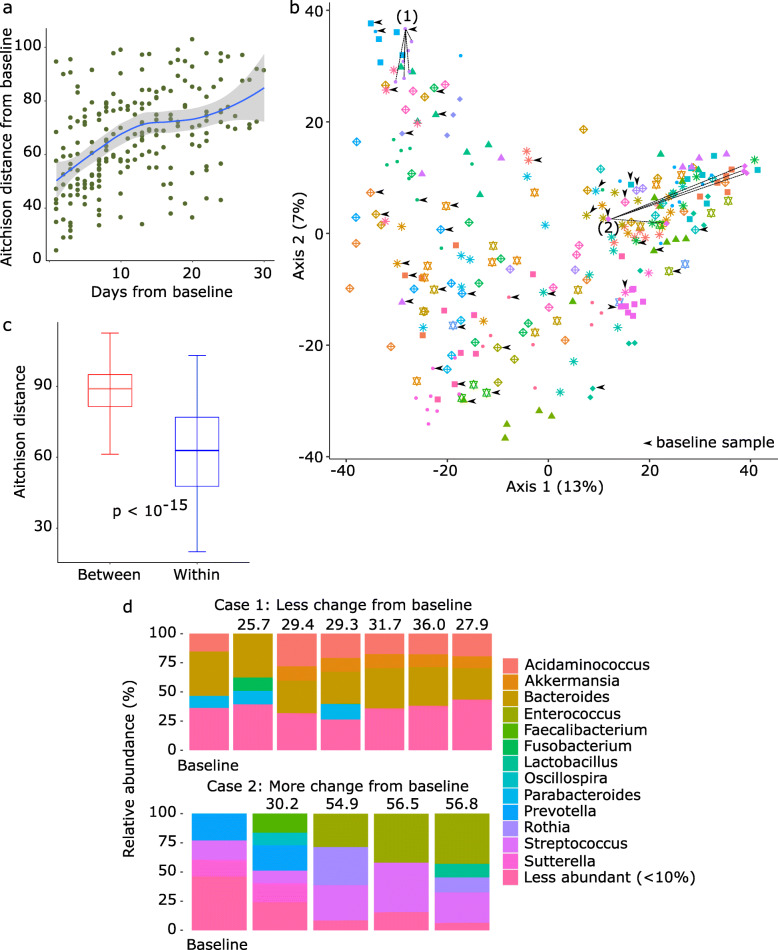
Microbiota variation among patients and over time. **a** Microbiota distance from baseline, as measured by Aitchison distance, vs. time. Time was measured in days relative to the baseline sample. The loess smoothed curve and its 95% confidence bar are shown. **b** Principal component analysis using Aitchison distance. Each point represents a sample, and points with the same color and shape represent samples from the same patient. Baseline samples are indicated by arrowheads. Numbers in parentheses indicate percent variation explained by the corresponding principal component. For two subjects (cases 1 and 2), non-baseline samples are connected to the baseline sample for the same patient. **c** Aitchison distance between samples collected from the same patient (“within”) vs. different patients (“between”). *p* value was calculated from a Wilcoxon test. **d** Distribution of taxa among the samples in the two cases (1 and 2) in **b**. Genera with relative abundances < 10% across all samples are grouped together. Baseline sample from each patient is shown on the left. The number above each non-baseline sample indicates its Aitchison distance from the corresponding baseline sample

The microbiota became more dissimilar to baseline over time (Fig. 3a). Despite this progressive departure from baseline, the greatest proportion of microbiota variance was explained by the individual (PERMANOVA *R*^2^ = 0.54, *p* < 0.001, adonis test with 999 permutations; Fig. 3b). Although interval from baseline, use of parenteral nutrition, and antibiotic history were also statistically significant in this analysis (*p* < 0.001), they only explained a small proportion of the variance (PERMANOVA *R*^2^ = 0.019, 0.007, and 0.006, respectively). Median Aitchison distance between samples from the same patient was significantly smaller than median distance between samples from different patients (*p* < 10^−15^; Wilcoxon test; Fig. 3c), indicating that microbial communities in samples from the same patient remained more similar to one another than to those from the other patients. As a potential mechanism for this host specificity, we evaluated whether baseline microbiota was a determinant of subsequent microbiota departures from baseline after controlling for antibiotics. Microbiota departure from baseline was denoted Δm and defined as the Aitchison distance [29] between baseline and non-baseline communities. We first quantified the antibiotic history of each sample by considering the time series of exposures to 7 major classes of antibacterial antibiotics between hospital admission and day the sample was collected. For a given day, if a given antibiotic was used, exposure to that antibiotic was coded 1 and otherwise coded zero. Next, we applied a decaying average function to the antibiotic history of each sample—a time series of 0s and 1s for each antibiotic class—to achieve a single numerical value summarizing the exposure history of a given sample for a given antibiotic. The decaying average method flexibly models both recent and less recent exposures by placing more weight on exposures in more recent days preceding the sample. Procrustes analysis (Additional file 1: Figure S2) showed that the correlation between microbiota composition and antibiotic history was robust to the specific choice of the decay factor. We chose a decay factor of 2 for our main analysis thereafter.

Next, we used principal component mixed effect regression including the top principal components (PCs) of antibiotic history for each non-baseline sample and the top PCs of the microbiota for the baseline sample from the same individual as predictors of Δm. This approach eliminates the problem of multicollinearity because the PCs are orthogonal. We included the first PC of the microbiota and the first PC of antibiotic history as predictors in the model. In addition, use of parenteral nutrition (categorical), interval from baseline (days), baseline Shannon index, and sample read depth were included as fixed effect covariates and patient ID as a random effect. The model was powerful, with a Pearson’s correlation coefficient comparing observed vs. predicted Δm values of 0.82 (Additional file 1: Figure S3a). Microbiota and antibiotic PCs and interval from baseline were significant predictors of Δm (Table 1). Although including more microbiota PCs would explain a larger proportion of microbiota variance, it did not improve performance of the regression model and the correlation coefficient remained 0.82. Model performance did not change when a slower decay (decay factor 1.1) was used to define antibiotic history (Additional file 1: Figure S3b). These findings indicate that baseline microbiota is a major independent determinant of Δm.

**Table 1:** Principal component mixed effect regression to evaluate whether baseline microbiota independently predicts Δm

Variable	Regression coefficient	Standard error	***p***
Baseline microbiota PC1	0.82	0.35	0.01
Antibiotic PC1	− 1.90	0.77	0.01
Interval from baseline (days)	0.98	0.12	< 0.01
Baseline Shannon index	− 5.70	4.19	0.19
Sample read depth	0.88	1.96	0.54
Parenteral nutrition	3.60	3.38	0.37

*PC* principal component; Δm, Aitchison distance between the baseline and subsequent microbiota

**Fig. 4:**
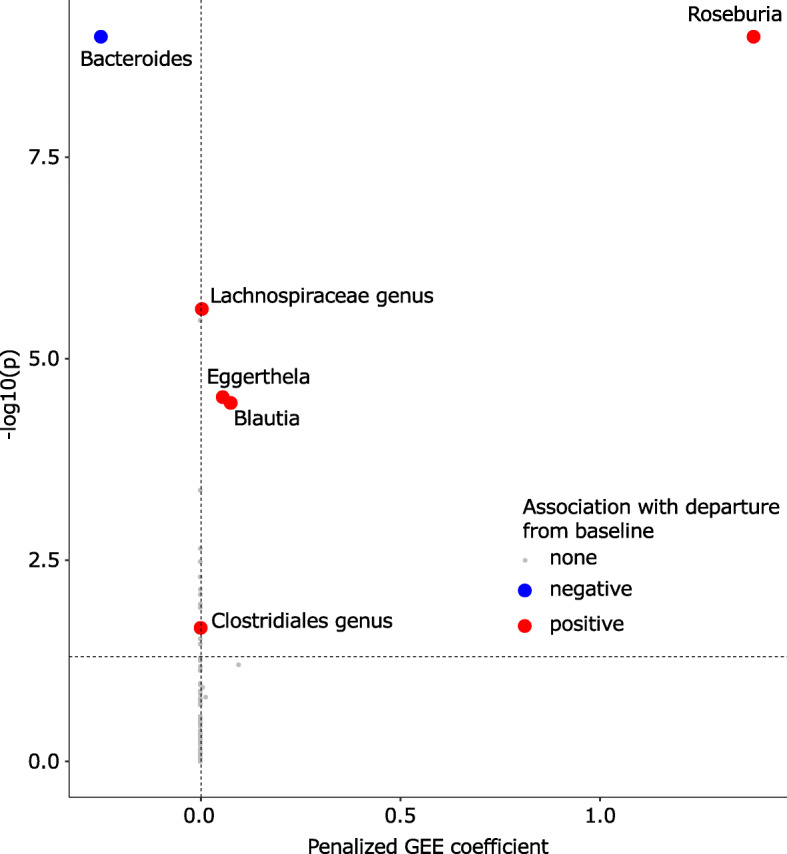
Baseline taxonomic predictors of microbiota departure from baseline. Volcano plot showing coefficients from penalized generalized estimating equations (PGEE) and their corresponding *p* values. The model included clr abundances of baseline OTUs collapsed at genus level, decay averaged antibiotic histories, age, sex, body mass index, use of parenteral nutrition, baseline Shannon index, sample depth, and interval from baseline as potential predictors of the Aitchison distance between non-baseline microbiota and baseline (pre-treatment) microbiota for the same patient. Patient ID was a random effect. The tuning parameter was 0.21, determined using fourfold cross validation. A first-order autoregressive correlation structure was assumed. The horizontal dashed line defines statistical significance (*p* < 0.05). Circles colored red or blue represent significant taxa with a coefficient > 10^−3^ (red) or < − 10^−3^ (blue), respectively. These taxa at baseline were associated with the extent of departure from baseline in subsequent samples and their corresponding circles were magnified for better visualization

To identify pre-treatment taxa with the largest contribution to Δm, we used the penalized generalized estimating equations (PGEE) method, which is suitable for longitudinal high-throughput data analysis [33, 34]. Features were baseline genus-level clr abundances (121 genera), decay averaged antibiotic histories (7 features), age, sex, body mass index, use of parenteral nutrition, baseline Shannon index, sample read depth, and interval from baseline. Patient ID was a random effect. Fourfold cross validation determined the optimal value of the tuning parameter to be 0.21. This value was then used to build the final PGEE model. In the final model, 5 baseline taxa (*Roseburia*, *Blautia*, *Eggerthella*, a Lachnospiraceae genus, and a Clostridiales genus) predicted larger Δm values, and one taxon (*Bacteroides*) predicted smaller Δm values (Fig. 4). None of the other variables predicted Δm. In a case study to demonstrate these findings (Fig. 3d), we chose two subjects: (*i*) case 1 with a relatively high abundance of *Bacteroides* in the baseline sample (34%) and (*ii*) case 2 with no *Bacteroides* in the baseline sample. As expected from PGEE results and with *Bacteroides* predicted to protect against change from baseline, subsequent samples from case 1 were less different from baseline (Aitchison distances 25.7–36.0) compared to subsequent samples from case 2 relative to baseline (Aitchison distances 30.2–56.8). This is also demonstrated in the PCA plot (Fig. 3b).

In post hoc exploratory analysis (Additional file 1 Table S1), we performed microfluidic quantitative PCR for antibiotic resistance genes (ARGs) in 27 longitudinal stool samples from 4 patients to understand whether obvious changes occur in longitudinal abundances of ARGs. The 3 most abundant ARGs were ermB (macrolide resistance, aggregate mean among samples: 6.57 copies/microL DNA), tetM (tetracycline resistance, 5.87 copies/microL DNA), and ermF (macrolide resistance, 5.30 copies/microL DNA). However, because antibiotics in these classes were not used in our patients, the ARGs likely did not play a role in our findings. vanA was the only ARG with clear dynamics in longitudinal samples. vanA confers vancomycin resistance to highly relevant pathogens in our patient population such as vancomycin-resistant enterococci. In two subjects, vanA was nearly or completely undetectable in the first week, then quickly rose in abundance in week 2, and remained high until the end of hospitalization.

## Discussion

We found for the first time that the pre-treatment state of human gut microbiota is a major determinant of antibiotic-induced microbiota perturbations. This finding in a clinical setting with intense antibiotic pressure suggests an even stronger predictive role for baseline microbiota when antibiotic exposure is less intense. Therefore, an important mechanism underlying individualized responses to antibiotics is the individualized microbiota at baseline. Considering that only a small fraction of gut microbiota is shared among healthy individuals [38, 39], our results explain the previous observations that the microbiota in different individuals responds differently to antibiotics [2, 40]. This is also consistent with the role of pre-treatment microbiota in determining response to fecal microbiota transplantation (FMT) [41] and dietary interventions [42].

We identified 6 pre-treatment taxa predictive of the magnitude of microbiota perturbation from baseline. *Roseburia*, *Blautia*, and *Eggerthella* predicted larger microbiota departures from baseline. Specific *Roseburia* species degrade dietary fiber β-mannan, producing short-chain fatty acids such as butyrate, with numerous and profound homeostatic effects on the host [43]. Specific compounds synthesized by bacteria can regulate the transcription of various genes within the community (quorum sensing) [44], potentially modulating microbiota susceptibility to antibiotics. Similarly, certain *Eggerthella* species have significant metabolic potential, contributing, for example, to the conversion of dietary fiber-derived lignans to bioactive compounds [45]. Whether and how this metabolic activity can impact the gut microbiota is unknown. Antimicrobial peptides produced by certain *Blautia* species have been shown to confer colonization resistance against antibiotic-resistant pathogens [46]. A microbial community with fewer antibiotic-resistant genes is expected to undergo larger perturbations after exposure to antibiotics. *Bacteroides* was the only genus with an apparent stabilizing effect. Quorum sensing has been well established for some species of this genus, which are abundant in the colonic microbiota (e.g., *Bacteroides fragilis*). Examples of quorum sensing mechanisms that could contribute to differential response to antibiotics include autoinducers of efflux pumps and biofilm formation [47]. Bacteroides species can also influence microbiota composition by secreting antimicrobial peptides in contact-dependent and -independent manners [48]. In addition, Bacteroides species are potent producers of propionate [49], which mediates colonization resistance against pathogens such as *Salmonella typhimurium* [50].

Considering the two phases of microbiota response to antibiotics, our study focused on phase 1, where the microbiota is perturbed under active antibiotic pressure. We included the characteristics of the antibiotic insult and baseline microbiota as the main potential determinants of phase 1 in our analysis. Although sex, age, and mode of nutrition were adjusted for, the role of other host factors such as genetics and immunity in phase 1 need to be evaluated in future research. Other potentially important variables include the specific type(s) of chemotherapy and proton pump inhibitors (PPIs). Chemotherapeutic regimens commonly used for patients with AML are not known to significantly alter gut microbiota, although an expansion of *Enterococcus* spp. and *Escherichia coli* has been reported [51]. In otherwise healthy adults and patients with inflammatory bowel disease, PPIs increased ectopic colonization of oral bacteria in the gut and promoted the expansion of *Enterococcus*, *Streptococcus*, *Staphylococcus*, and some *Proteobacteria* [52, 53]. We use PPIs in our patients only if they develop significant upper gastrointestinal side effects from chemotherapy. In our experience, such patients typically require parenteral nutrition, a variable included in our models. To avoid anticipated multicollinearity, we did not consider PPI use as a separate variable. Studies with larger sample sizes would permit building more comprehensive models incorporating a larger number of variables. In addition, phase 2 (recovery) is a critical segment of the microbiota trajectory that needs to be studied further. Finally, how the two phases are interrelated and contribute to clinical outcomes is unknown. It is unclear, for example, whether robust microbiota recovery after perturbation towards baseline might compensate for a large initial departure from baseline. Also, whether a larger departure from baseline predicts a smaller chance for full recovery is unknown.

In conclusion, knowledge about pre-treatment microbiota can be used to predict the magnitude of antibiotic-induced perturbations and lead to personalized precision therapeutics. We identified specific taxa in the pre-treatment microbiota that may predict the extent of antibiotic perturbation. Our findings lead to hypothesis generation for testing and validation in future studies. Mechanisms by which specific taxa in gut microbiota communicate with other members of this complex community to regulate stability, resistance, and resilience is a subject of intense investigation. Quorum sensing and horizontal transfer of ARGs are two possible mechanisms. Our preliminary results on ARGs suggest potentially important ARGs with dynamics that could influence microbiota response to antibiotics over time. Finally, the patient population in this study was one with aggressive cancer receiving chemotherapy in a nosocomial setting and with altered nutrition. The extent to which our findings may be generalized to other clinical settings with antibiotic exposure remains to be determined.

## Supplementary Information

**Additional file 1: Figure S1** Taxonomic distribution in baseline and non-baseline samples. (a) Mean relative abundance of the most abundant taxa are shown. The top 15 taxa in each group were used to generate the plot. (b) Linear discriminant analysis Effect Size using an LDA score threshold of 3.0 and p value threshold of 0.05. The deepest level of taxonomy was genus (g), and taxa unclassifiable at the genus level are shown at family (f) or order (o) level. **Figure S2** Procrustes analysis correlating microbiota composition with antibiotic history. (a) Decay factor 2.0. (b) Decay factor 1.5. (c) Decay factor 1.1. **Figure S3**. Principal component mixed effect regression. The first PC of microbiota for the baseline sample, first PC of antibiotic history for the non-baseline sample, baseline Shannon diversity, read depth of the non-baseline sample, use of parenteral nutrition (categorical) before the non-baseline sample, and the time interval in days between the two samples were included as fixed effect predictors of Aitchison distance between the two samples. Patient ID was a random effect. Model performance was defined as the Pearson’s correlation coefficient (r) comparing observed vs. predicted values of the outcome variable. Different values of the decay factor were used in the two panels. PC: principal component. **Table S1** Microfluidic quantitative PCR of antibiotic resistance genes.

## Data Availability

Sequence data were deposited in the Sequence Read Archive of the National Center for Biotechnology’s Information (NCBI) under BioProject ID SRP141394.
