# Supplementary material for: Gut microbiota response to antibiotics is personalized and depends on baseline microbiota
Source: Microbiome. 2021 Oct 27;9:211. doi: 10.1186/s40168-021-01170-2 (PMC8549152; doi:10.1186/s40168-021-01170-2)
Supplement: Supplementary file 2 — Additional file 1: Figure S1 Taxonomic distribution in baseline and non-baseline samples. (a) Mean relative abundance of the most abundant taxa are shown. The top 15 taxa in each group were used to generate the plot. (b) Linear discriminant analysis Effect Size using an LDA score threshold of 3.0 and p value threshold of 0.05. The deepest level of taxonomy was genus (g), and taxa unclassifiable at the genus level are shown at family (f) or order (o) level. Figure S2 Procrustes analysis correlating microbiota composition with antibiotic history. (a) Decay factor 2.0. (b) Decay factor 1.5. (c) Decay factor 1.1. Figure S3. Principal component mixed effect regression. The first PC of microbiota for the baseline sample, first PC of antibiotic history for the non-baseline sample, baseline Shannon diversity, read depth of the non-baseline sample, use of parenteral nutrition (categorical) before the non-baseline sample, and the time interval in days between the two samples were included as fixed effect predictors of Aitchison distance between the two samples. Patient ID was a random effect. Model performance was defined as the Pearson’s correlation coefficient (r) comparing observed vs. predicted values of the outcome variable. Different values of the decay factor were used in the two panels. PC: principal component. Table S1 Microfluidic quantitative PCR of antibiotic resistance genes. [file 40168_2021_1170_MOESM2_ESM.pdf]

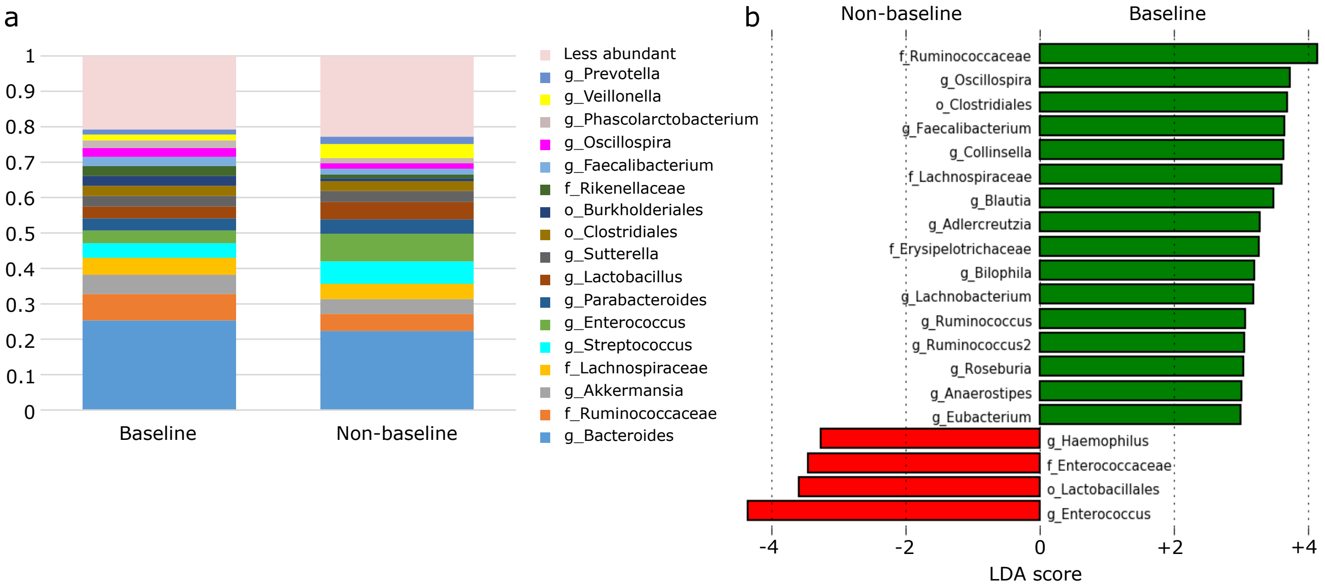

Figure S1: Taxonomic distribution in baseline and non-baseline samples
(**a**) Mean relative abundance of the most abundant taxa are shown. The top 15 taxa in each group were used to generate the plot. (**b**) Linear discriminant analysis Effect Size using an LDA score threshold of 3.0 and *p* value threshold of 0.05. The deepest level of taxonomy was genus (g). Taxa unclassifiable at the genus level are shown at family (f) or order (o) level.

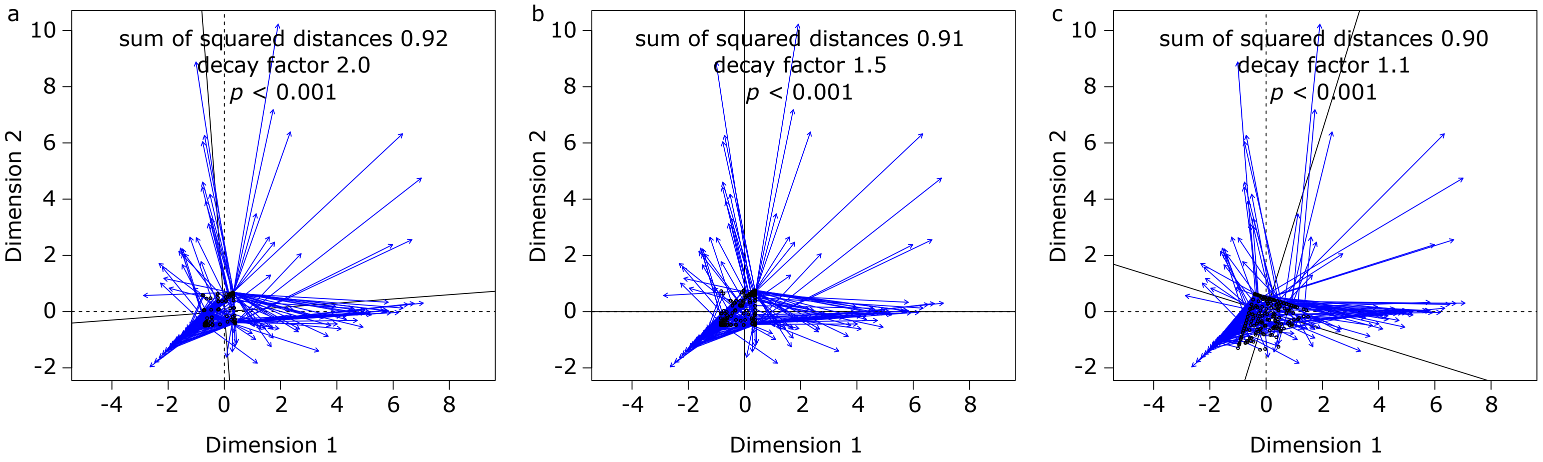

Figure S2: Procrustes analysis correlating microbiota composition with antibiotic history
(**a**) Decay factor 2.0. (**b**) Decay factor 1.5. (**c**) Decay factor 1.1.

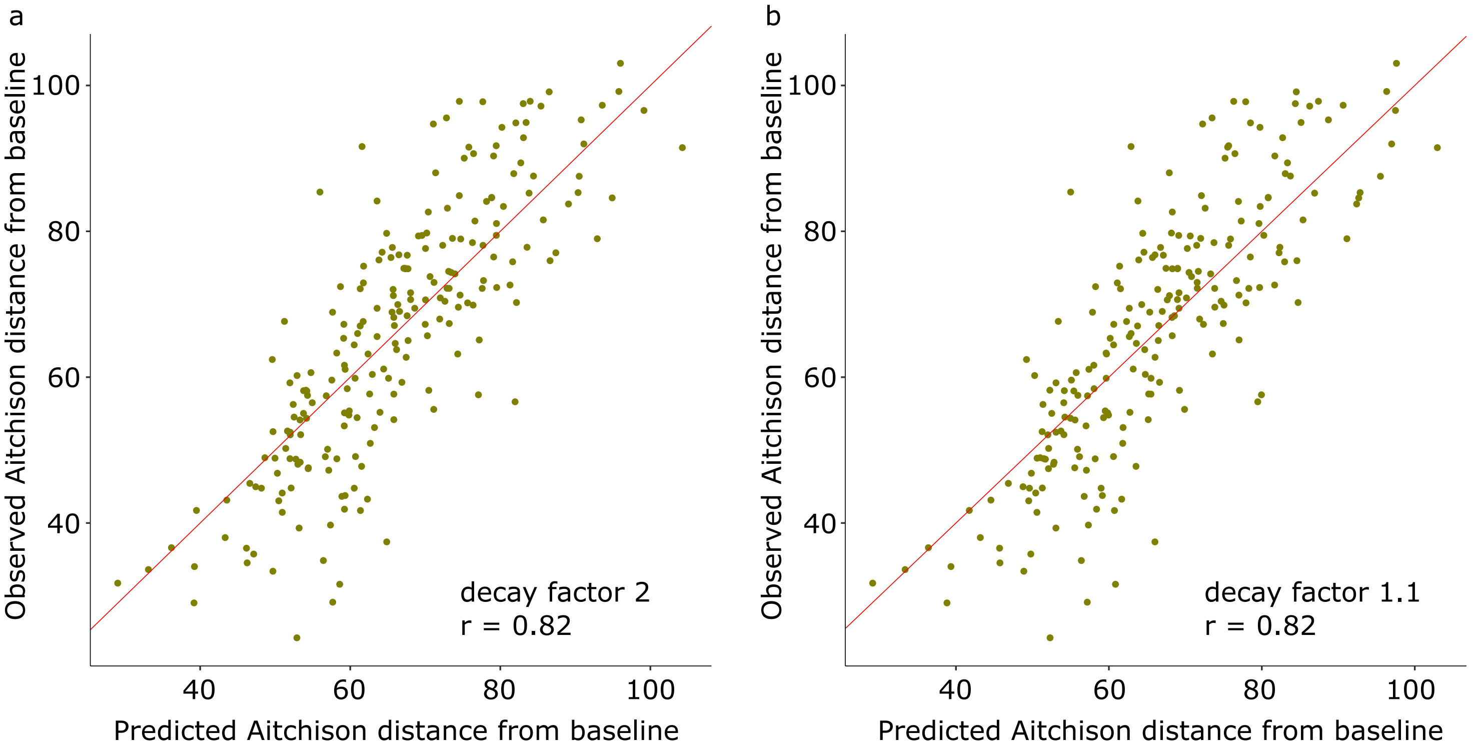

Figure S3: Principal component mixed effect regression
The first PC of microbiome for the baseline sample, first PC of antibiotic history for the non-baseline sample, baseline Shannon diversity, read depth of the non-baseline sample, use of parenteral nutrition (categorical) before the non-baseline sample, and the time interval in days between the two samples were included as fixed effect predictors of Aitchison distance between the two samples. Patient ID was a random effect. Model performance was defined as Pearson's correlation coefficient (r) comparing observed vs. predicted values of the outcome variable. Different values of the decay factor were used in the two panels. PC: principal component

Supplementary Table S1: Microfluidic quantitative PCR of antibiotic resistance genes

DNQ (detected but not quantifiable): values lower than the lowest concentration of the standard curves; ND (not detected)

			Aminoglycosides						Beta-lactams											Macrolides				Quinolones		Sulfonamides			Tetracyclines						Glycopeptides		Ansamycins	Diaminopyrimidine	Chloramphenicol		
**Subject**	**Day**	**16S**	**aacA**	**aadA5**	**aadD**	**acrD**	**mexB**	**strB**	**ampC**	**bla_CTX-M32**	**bla_imp13**	**bla_KPC**	**bla_NDM1**	**bla_NPS**	**bla_OXA**	**bla_SHV**	**bla_VIM**	**merA**	**ereB**	**ermB**	**ermF**	**mefE**	**qnrA**	**qnrB**	**sul1**	**sul2**	**sul3**	**tetA**	**tetL**	**tetM**	**tetS**	**tetW**	**tetX**	**vanA**	**vanB**	**arr2**	**dfr13**	**cmlB**	**floR**	**catB8**
21	4	5.72	ND	1.30	1.32	1.29	3.87	2.38	1.11	ND	ND	4.52	1.53	ND	DNQ	5.14	0.19	4.05	DNQ	7.17	5.37	5.81	1.32	ND	1.86	1.70	1.33	2.64	1.62	7.49	3.88	7.08	1.73	ND	2.91	0.58	ND	DNQ	2.50	ND
21	7	5.66	0.58	1.46	1.83	DNQ	3.96	0.89	1.00	ND	ND	5.01	1.44	ND	ND	5.00	0.87	4.11	ND	7.15	6.00	5.64	0.85	DNQ	1.04	ND	0.93	2.48	1.55	7.12	3.96	7.10	DNQ	0.84	3.09	1.05	ND	0.51	2.13	ND
21	8	4.76	ND	0.99	1.72	ND	2.98	ND	1.43	ND	ND	6.21	ND	ND	ND	4.30	DNQ	2.80	ND	6.49	3.73	1.28	1.02	ND	1.84	ND	0.41	1.95	1.46	6.28	3.84	5.65	2.60	1.56	2.98	ND	ND	0.44	0.56	ND
21	11	5.73	ND	0.86	1.86	ND	3.46	ND	1.77	ND	ND	4.47	ND	ND	ND	4.41	DNQ	3.07	ND	7.18	5.23	2.23	0.55	ND	1.46	ND	0.61	2.27	0.50	7.51	3.88	6.92	ND	ND	3.28	0.23	0.34	0.91	1.53	ND
21	14	5.13	ND	1.06	0.78	ND	ND	ND	1.46	ND	ND	6.91	ND	ND	0.43	4.27	DNQ	3.28	DNQ	6.73	4.86	0.64	0.82	ND	ND	ND	0.68	2.78	1.69	6.97	3.13	6.50	DNQ	0.52	2.73	0.09	ND	0.77	1.22	ND
21	19	5.00	ND	1.06	ND	0.04	3.37	DNQ	1.29	ND	ND	1.22	1.74	ND	ND	4.62	DNQ	3.04	ND	7.14	2.84	0.87	1.26	ND	ND	ND	0.72	2.60	1.37	6.98	2.62	6.50	ND	ND	2.77	0.42	ND	DNQ	1.81	ND
21	22	4.66	ND	1.18	0.38	ND	ND	ND	1.77	ND	ND	6.62	1.64	ND	1.37	4.58	DNQ	2.05	ND	6.85	3.56	0.74	0.97	ND	1.50	ND	0.62	2.47	1.49	6.60	1.55	6.12	ND	ND	2.30	0.80	ND	0.84	1.99	ND
21	28	5.01	ND	1.24	0.61	ND	ND	ND	2.04	ND	ND	4.81	ND	ND	ND	3.51	ND	1.97	ND	6.67	5.15	ND	ND	ND	ND	ND	0.69	2.08	0.01	6.85	2.84	6.60	ND	ND	2.68	ND	ND	0.68	0.18	ND
21	33	5.80	ND	0.65	2.94	ND	2.01	1.15	1.09	2.11	ND	4.03	ND	ND	ND	4.30	ND	3.26	ND	6.83	3.84	3.90	0.82	ND	ND	1.35	0.66	2.06	1.88	6.71	2.87	6.98	ND	ND	3.38	0.17	ND	DNQ	1.40	ND
22	0	4.64	ND	0.93	0.55	3.47	2.77	0.30	2.69	ND	0.50	4.18	ND	ND	ND	4.39	ND	3.61	ND	7.28	7.41	3.66	1.40	ND	ND	1.44	0.91	1.15	1.82	6.04	ND	5.83	7.23	ND	DNQ	0.79	0.97	ND	1.89	ND
22	3	4.79	ND	0.87	0.52	5.75	ND	2.09	4.94	ND	ND	6.59	0.75	ND	ND	4.34	0.50	1.71	ND	7.48	7.41	3.84	DNQ	0.33	1.25	ND	1.00	1.52	DNQ	6.00	1.49	6.03	6.96	ND	1.43	ND	ND	ND	DNQ	ND
22	7	4.60	ND	0.43	2.37	3.31	ND	2.07	2.55	ND	1.07	6.44	ND	ND	ND	3.98	ND	3.35	ND	6.99	6.95	3.43	ND	ND	2.05	ND	0.66	1.43	ND	5.87	2.79	5.80	6.85	ND	1.21	ND	ND	0.51	ND	ND
22	10	4.26	ND	1.24	2.76	ND	ND	1.50	1.00	ND	ND	6.02	1.38	ND	DNQ	3.69	ND	ND	ND	7.21	7.41	2.81	ND	ND	ND	ND	0.88	2.18	0.80	5.95	DNQ	5.27	6.88	ND	1.40	ND	DNQ	DNQ	1.31	ND
22	13	4.18	ND	0.69	4.01	ND	ND	ND	2.12	1.90	ND	2.66	ND	ND	ND	3.90	ND	2.31	ND	5.73	ND	0.37	ND	ND	1.40	ND	0.94	DNQ	2.12	7.85	1.34	1.17	1.85	6.64	1.59	ND	ND	ND	ND	ND
22	17	3.53	ND	DNQ	2.83	ND	ND	1.39	1.01	ND	ND	2.85	ND	ND	ND	0.84	ND	2.06	ND	6.21	ND	ND	ND	ND	1.52	ND	0.04	1.90	ND	7.20	0.59	2.15	ND	6.20	DNQ	ND	ND	ND	ND	ND
22	24	3.45	ND	1.10	3.82	ND	ND	1.19	1.62	ND	ND	3.54	ND	ND	ND	3.19	ND	2.67	ND	4.30	3.69	1.49	ND	ND	ND	ND	0.51	2.07	ND	7.29	3.03	2.56	0.54	6.43	ND	ND	ND	ND	0.44	ND
22	20	3.56	ND	0.72	3.32	ND	3.27	1.44	1.24	1.59	ND	3.11	1.19	ND	ND	3.27	ND	2.36	ND	5.59	ND	ND	ND	ND	1.50	ND	DNQ	ND	ND	7.24	2.43	2.10	ND	6.19	ND	ND	ND	ND	1.79	ND
22	27	4.76	ND	0.91	4.25	ND	ND	2.32	0.63	ND	ND	3.84	ND	ND	ND	3.25	ND	2.79	ND	7.21	0.02	3.06	ND	ND	1.19	ND	DNQ	2.43	ND	7.75	2.80	2.92	ND	6.64	0.56	ND	ND	ND	ND	ND
23	1	5.22	0.26	0.80	2.04	3.14	3.35	5.10	2.89	ND	ND	4.65	1.66	ND	0.99	4.69	1.79	3.22	ND	7.21	7.43	4.99	DNQ	ND	1.07	5.04	0.63	2.01	1.66	5.14	2.29	6.75	6.12	0.36	2.46	0.57	ND	ND	1.99	ND
23	2	5.52	ND	1.02	1.53	2.65	2.37	5.18	2.19	ND	ND	4.56	0.70	ND	0.91	4.75	2.52	3.18	ND	7.16	7.46	4.91	0.91	ND	1.72	5.14	1.10	2.46	0.97	5.00	3.08	6.81	6.94	DNQ	2.14	ND	ND	0.39	1.71	ND
23	5	4.10	ND	0.61	0.85	1.69	2.75	3.87	1.74	ND	ND	4.78	0.44	0.81	0.04	4.50	1.91	3.09	ND	6.76	7.33	4.43	0.67	0.74	1.44	3.88	0.81	2.46	1.26	4.45	1.89	6.37	6.71	ND	1.77	ND	ND	1.20	1.64	ND
23	9	5.31	0.48	1.44	2.61	0.91	2.80	2.54	0.96	1.52	ND	4.63	1.27	ND	0.03	5.03	2.42	3.84	ND	7.18	7.96	4.78	DNQ	1.04	DNQ	ND	1.44	2.51	2.93	6.00	1.79	6.63	7.49	5.39	0.95	DNQ	1.34	ND	1.04	ND
23	12	3.72	ND	1.97	3.31	ND	3.71	ND	1.34	ND	ND	3.49	1.31	ND	ND	3.91	ND	2.01	ND	6.81	6.98	3.19	ND	ND	1.36	ND	0.09	DNQ	2.88	2.91	DNQ	5.28	6.50	6.33	ND	ND	ND	ND	ND	0.80
23	16	4.12	ND	0.97	2.56	ND	1.71	ND	1.63	ND	ND	5.44	ND	ND	DNQ	3.65	ND	ND	ND	6.58	6.24	2.66	0.26	ND	ND	ND	0.70	2.74	2.72	3.05	ND	4.73	5.56	5.98	0.41	0.17	ND	ND	1.54	ND
23	19	2.77	ND	1.29	1.66	ND	ND	0.83	2.33	ND	ND	2.67	ND	ND	ND	1.85	ND	ND	ND	5.91	2.73	0.52	0.98	ND	ND	ND	0.21	2.71	ND	3.02	2.02	2.28	0.96	5.21	ND	0.23	ND	ND	1.22	ND
23	23	2.22	ND	1.54	1.26	ND	ND	ND	1.04	ND	ND	2.92	0.90	ND	0.77	4.12	0.89	2.97	ND	4.93	2.93	DNQ	ND	ND	ND	ND	0.81	2.95	ND	2.90	2.81	1.91	ND	4.03	0.95	DNQ	ND	0.89	1.04	ND
24	2	2.32	ND	0.65	1.14	ND	ND	1.10	1.49	ND	ND	4.40	ND	ND	ND	1.82	ND	2.32	ND	4.52	4.72	2.05	ND	ND	0.90	1.12	0.19	ND	ND	2.42	ND	3.63	3.85	1.62	ND	ND	ND	0.91	ND	ND
